# Dietary patterns in the healthy oldest old in the healthy aging study and the Canadian longitudinal study of aging: a cohort study

**DOI:** 10.1186/s12877-020-01507-w

**Published:** 2020-03-16

**Authors:** Qianqian Gu, Carly M. Sable, Angela Brooks-Wilson, Rachel A. Murphy

**Affiliations:** 1 School of Population and Public Health, University of British Columbia, 2206 East Mall, Vancouver, BC V6T 1Z3 Canada; 2 Canada’s Michael Smith Genome Sciences Centre, BC Cancer, Vancouver, BC Canada; 3 Department of Biomedical Physiology and Kinesiology, Simon Fraser University, Burnaby, BC Canada; 4 Cancer Control Research, BC Cancer, Vancouver, BC Canada

**Keywords:** Aging, Nutrition, Longevity, Centenarians

## Abstract

**Background:**

Very few people live to eighty-five years and older (the ‘oldest old’), and even fewer live to this age without developing chronic diseases. It is important to understand the relationship, if any, of modifiable factors such as diet on healthy aging. However, there are few studies of diet among healthy oldest old, especially in North American populations. We aimed to characterize dietary patterns among ‘super-seniors’ (SS) within the Canadian Healthy Aging Study.

**Methods:**

122 SS aged 85 years or older and free of cancer, cardiovascular or pulmonary disease, dementia and diabetes were recruited. Comparisons were made to 12,626 participants aged 65–86 in the Canadian Longitudinal Study of Aging who completed the same 36-item food frequency questionnaire that queried consumption over the prior 12 months of nutrients and foods thought to be important for aging. Dietary patterns were identified with principal component analysis. The odds of being a SS were determined for quartiles of each dietary pattern with logistic regression.

**Results:**

Two dietary patterns were identified; a western diet characterized by french fries, red meat, processed meat and a nutrient-rich diet which included fruits, vegetables, whole grains, nuts and seeds among other healthy food choices. Higher scores for both dietary patterns were associated with increased odds of being a SS, however, only the western dietary pattern remained associated with adjustment for covariates (Quartile 4: OR = 3.21, 95% CI 1.91–5.51).

**Conclusions:**

Our finding adds to the limited evidence on dietary intake among the healthiest oldest old but it is unclear whether assocations reflect generational differences between groups or possible contributions to longevity.

## Background

Broadly, healthy aging refers to development and maintenance of the functional ability to enable wellbeing [1]. The emphasis of healthy aging is on healthspan not lifespan with some definitions also emphasizing aging in the absence of disease [2]. The oldest old, those who are eighty-five years and older [3] are among the fastest growing segment of the population in some parts of the world [4]. Few people however, live to this age without developing chronic diseases. It is thus important to understand the influence of modifiable lifestyle factors such as diet on the achievement of exceptional longevity and the role, if any, of these factors on healthspan.

Several studies among long-lived populations have yielded important insight into dietary patterns and nutrients associated with morbidity or life-span. Long-term caloric restriction and traditional dietary patterns such as the Okinawan diet and Mediterranean diet have been found to be potentially important for reducing age-related diseases and supporting longevity [5, 6]. Some studies have compared dietary intake and diet patterns of centenarians to those of ‘younger’ adult cohorts from the same geographical region [7–12]. Such studies generally report lower energy and/or fat intake for the centenarians as compared to community-dwelling older adults in their 60s or 80s. Conversely, centenarians have also been observed to consume more high-fat foods, and were less likely to avoid dietary cholesterol [10, 13].

There are in relative terms, few studies of the oldest old and centenarians in gerontology research, and even fewer studies of populations that have survived to that age in the absence of a major age-related chronic disease. Studies that characterize dietary intake in such populations will add to our knowledge base and provide critical information for hypothesis testing. Our aim was thus to assess the dietary intake of a population of men and women 85 years and older who were free of chronic diseases and compare dietary patterns to adults aged 65 to 86 years using data from a Canada -wide population-based study of aging. We hypothesized that compared to the younger cohort, older healthy adults would have dietary patterns that more closely follow guidelines for chronic disease risk reduction such as more frequent consumption of fruits and vegetables, whole grains and lean protein.

## Methods

### Study participants-healthy aging study

This study was nested within the Healthy Aging Study [14–17] that was designed to study genetic factors that underlie healthy aging and resistance to age-related diseases. Between 2004 and 2007, participants 85 years and older from Metro Vancouver, British Columbia, Canada were recruited as described in detail previously [17]. Eligibility criteria for super-seniors (SS) included age 85 and older, self-reporting never been diagnosed with or taking medication prescribed for cancer (excluding non-melanoma skin cancer), cardiovascular or pulmonary disease (excluding asthma), dementia or diabetes.

### Canadian longitudinal study on aging (CLSA)

The CLSA is a national longitudinal study of 50,000 Canadians aged 45 to 85 years [18]. The Canadian Community Health Survey (CCHS) was used to recruit participants into the CLSA. The sampling frame is nationally representative, although residents in the Northwest Territories, Nunavut, Yukon, some remote regions, persons living on federal First Nations reserves and other First Nations settlements, full-time members of the Canadian Armed Forces and individuals living in institutions were excluded. A subset of 30,000 participants (the “Comprehensive Cohort”) underwent in-depth face to face interview questionnaires which included dietary assessment. Baseline data was collected over a 3 year period and completed in June 2015. To provide a large, comparative group for SS’s, data was obtained from CLSA participants with dietary information along with covariates that mirrored those collected in our study of SS. Our analysis was confined to CLSA participants aged 65 years and older which provided a comparative group of 12,626 older adults. To most closely approximate the general aging population in Canada, no addition exclusion criteria were applied to the CLSA dataset.

### Dietary intake

Dietary information was collected using the same 36-item Short Diet Questionnaire (SDQ) that was developed and used in the CLSA to assess usual consumption frequencies (last 12 months) of key nutrients and foods of importance for health promotion and chronic disease prevention in younger and older adults [19]. The SDQ has been tested for use in community-dwelling older adults (70 years and older) in a subsample of the Québec Longitudinal Study on Nutrition and Successful Aging (NuAge) Study [19]. The SDQ was selected for use in this study to enable direct dietary comparisons with a large national sample of older adults. The SDQ has been validated relative to three 24-h diet recalls and has been shown to be a reasonable approach to obtain usual frequencies of food sources of fats, regular and low-fat food choices, fibre, calcium, vitamin D, whole grains, calcium-fortified foods and beverages, and fruits and vegetables [19]. Responses to questions were given as frequency, e.g. number of times food or drink was consumed per day, week or month. Questions about portion size were not included in the SDQ.

### Data collection-SS

Recruitment packages were mailed in January and February 2017 to 177 SS participants who had consented to be re-contacted for further research. Participants were asked to complete a questionnaire on demographics and dietary intake by April 2017. From the potential pool of 177 participants, 2 were not interested in the study, 4 were deceased, 2 had moved, 20 did not respond, and 14 were living in assisted care. Due to the time lapse between recruitment for the Healthy Aging Study and this study, participants were asked whether they had chronic disease: cancer, diabetes, cardiovascular or heart disease, stroke or ischemic attack, peripheral vascular disease, lung disease or emphysema, dementia or Alzheimer’s disease, with response options of yes, no, don’t know or refused. Participants who answered ‘yes’ to any of the queried incident diseases were excluded (*N* = 8). A further five participants were excluded as they were unable to be subsequently reached to clarify a substantial amount of missing data on the SDQ (32 to 36/36 questions) as well as covariates that would have informed imputation (e.g. sex). The final sample size of SS was 122. Of the included participants, 2 completed the questionnaire via telephone.

Demographic questions were drawn from the CCHS [20] and included date of birth, sex, marital status, education, ethnicity, income, history of smoking daily, current smoking status, history of alcohol consumption, frequency of alcohol consumption in the prior 12 months, and self-reported height and weight.

### Statistical analysis

Characteristics were compared between groups with two-sided t-tests, chi-square tests or Fisher’s exact test. The 36 nutrients and foods were pre-assigned to 21 food groups based on nutrient profiles. Data was cubic root transformed to meet normality. A small proportion of participants had missing dietary data. To maximize our sample size, data were imputed with the mean frequency of intake of the same sex from the same group (SS or CLSA). Principal component analysis was performed to reduce food groups into a smaller set of variables and identify dietary patterns. An orthogonal varimax rotation was used. Scree plots were used to determine the number of factors to retain. A component loading of 0.3 or higher was contained within each factor, forming a dietary pattern. Factor loadings represent the relationship of each food or food group to the underlying factor. Each participant was assigned a factor score for each dietary pattern, and then grouped into quartiles. Participants in quartile 4 of a given diet pattern had the greatest tendency to follow the diet.

Multivariable logistic regression was used to calculate the odds ratios (ORs) and 95% confidence intervals (CIs) for being a SS. Quartile 1 was the reference for each dietary pattern. Model 1 was unadjusted. The change in coefficient method was used to determine significant confounders. In this procedure, variables were added to the model and remained in the model if they changed the coefficient by at least 10%. Model 2 was adjusted for sex, ethnicity (white, other ethnicity), marital status (living without a partner, living with a partner), household income (<$50,000, $50,000–$99,999, ≥$100,000), education (≤high school, >high school but <Bachelor’s degree, ≥Bachelor’s degree), smoking status (never smoker, former smoker, current smoker), alcohol consumption (never, occasional:<one a month, about once a month, 2–3 times a month, regular: once a week, 2–3 times a week, habitual: 4–5 times a week, or almost every day) and BMI (underweight: < 18.5 kg/m^2^, normal weight: 18.5–24.9 kg/m^2^, overweight: 25.0–29.9 kg/m^2^, obese: ≥30 kg/m^2^). Models were not adjusted for age as there was minimal overlap between SS and CLSA.

Statistical significance was determined at α < 0.05. All analyses were performed with R version 3.4.3 (R Foundation for Statistical Computing, Vienna, Austria).

## Results

Table 1 presents the descriptive statistics of 122 SS and 12,626 CLSA participants. A smaller proportion of SS participants lived with partners (37% versus 62%, *p* < 0.01) and had a Bachelor’s degree or higher (37% versus 49%) compared to CLSA participants. Never smokers were more common among SS (53% versus 43%) and current smokers were less common (0% versus 5.6%) compared to CLSA participants. Differences were also observed for alcohol consumption and BMI, whereby regular drinking and habitual drinking were less and more prevalent in SS than CLSA, respectively. The prevalence of normal BMI (63% versus 28%) was higher among SS participants while the prevalence of overweight and obese BMI were lower among SS (overweight: 28% versus 43% and obese: 4.1% versus 28%) compared to CLSA participants.

**Table 1:** Characteristics of CLSA participants and super-seniors (SS) in the study sample

	CLSA ***N*** = 12,626	SS ***N*** = 122	***p***-value
**Age, Mean (SD)**	73.1(5.7)	90.4 (3.6)	< 0.001
**Sex, N (%)**			0.08
Male	6329 (50.1)	51 (41.8)	
Female	6297 (49.9)	71 (58.2)	
**Ethnicity, N (%)**			0.45
White	12,191 (96.6)	120 (98.4)	
Other ethnicities	435 (3.4)	2 (1.6)	
**Marital status, N (%)**			< 0.001
Living without partner	4759 (37.7)	77 (63.1)	
Living with partner	7866 (62.3)	45 (36.9)	
**Household income, N (%)**			0.86
< $50,000	4717 (41.0)	50 (43.5)	
$50,000-99,999	4529 (39.4)	43 (37.4)	
≥ $100,000	2257 (19.6)	22 (19.1)	
**Education, N (%)**			0.01
≤ high school	2542 (20.2)	35 (28.7)	
> high school, <Bachelor’s degree	3858 (30.6)	42 (34.4)	
≥ Bachelor’s degree	6188 (49.2)	45 (36.9)	
**Smoking, N (%)**			0.001
Never smoker	5461 (43.3)	65 (53.3)	
Former smoker	6451 (51.1)	57 (46.7)	
Current smoker	713 (5.6)	0 (0.0)	
**Alcohol (past 12 months), N (%)**			< 0.001
Never	1640 (13.4)	16 (13.1)	
Occasional	3608 (29.4)	29 (23.8)	
Regular	3250 (26.5)	19 (15.6)	
Habitual	3773 (30.7)	58 (47.5)	
**BMI, N (%)**			< 0.001
Underweight	106 (0.8)	6 (5.0)	
Normal	3529 (28.1)	76 (62.8)	
Overweight	5388 (42.9)	34 (28.1)	
Obese	3543 (28.2)	5 (4.1)	

Principal component analysis identified two factors that explained 23% of the total variance. Foods contained in each factor and factor loadings are shown in Table 2. The first factor was characterized by western foods. The strongest associations for factor 1 were processed meat (factor loading 0.62), red meat (0.59), sauces and gravies (0.58), fried potatoes (0.54) and non-fried potatoes (0.54). Associations between factor 1 and high-sugar snacks, high-fat dairy, butter and margarine and salty snacks also exceeding factor loading thresholds (> 0.30). The second factor was characterized by nutrient-rich foods including fruits (0.61), non-starchy vegetables (0.69), whole grains (0.46), legumes (0.45), nuts and seeds (0.46), low-fat dairy products (0.31), salad dressing (0.37) and fish (0.49). The factors are hereafter referred to as ‘western dietary factor’ and ‘nutrient-rich dietary factor’.

**Table 2:** Factor loading for the western and nutrient-rich dietary patterns

	Factor 1	Factor 2
	Loading strength	Loading strength
Processed meat	0.62	−0.07
Red meat	0.59	−0.01
Sauces and gravies	0.58	0.00
Potatoes (fried)^1^	0.54	−0.21
Potatoes (non-fried)^2^	0.54	0.06
Butter and margarine	0.46	−0.03
High sugar snacks^3^	0.43	0.04
Salty snacks	0.38	−0.05
High fat dairy	0.31	0.06
Salad dressing	0.27	0.37
Poultry	0.25	0.25
Fruit juice	0.24	0.02
Eggs	0.19	0.25
Whole grains	0.03	0.46
Low fat dairy	−0.01	0.31
Legumes	−0.03	0.45
Fish	−0.05	0.49
Nuts and seeds	−0.07	0.46
Non-starchy vegetables	−0.07	0.69
Fruits	−0.19	0.61
Calcium-fortified foods^4^	−0.22	0.15

^1^french fries, poutine or other fried potatoes, ^2^boiled, mashed or baked potatoes, ^3^chocolate, cakes, pies, doughnuts, pastries, cookies, muffins, ice-cream, ice milk, frozen yogurt, milk-based desserts, ^4^calcium fortified foods, calcium fortified juice, calcium fortified milk and other calcium fortified beverages

Table 3 shows the unadjusted and adjusted ORs and 95% CIs for being a SS based on logistic regression. The highest quartile of the western dietary factor was associated with greater odds of being a SS (Model 1, Q4 OR = 2.06, 95% CI 1.29–3.38). After adjustment for confounders, associations were strengthened (Model 2, Q4 adjusted OR = 3.21, 95% CI 1.91–5.51). No associations were observed for quartiles 2 or 3. The highest quartile of the nutrient-rich dietary factor was associated with greater odds of being a SS in the unadjusted model (Model 1, Q4 OR = 1.75, 95% CI 1.10–2.85). Associations were attenuated in Model 2 after adjustment for covariates (Q4 OR = 1.57, 95% 0.95–2.66). No associations were observed for quartiles 2 or 3 in either model. Variables that were associated with group (SS or CLSA) and dietary pattern included sex, ethnicity, marital status, household income, education, smoking status, alcohol consumption and BMI). Of these, smoking, BMI and alcohol consumption had the largest impact on effect estimates. Covariate estimates are shown in Additional file 1: Table 1.

**Table 3:** Odds ratios (OR) of being a super-senior participant for the western and nutrient-rich dietary factors, results from unadjusted and adjusted logistic regression models among 12,626 CLSA and 122 SS

	Model 1		Model 2	
	OR (95% CI)	p-value	OR (95% CI)	p-value
**Western dietary factor**				
Q1	1.00		1.00	
Q2	0.88 (0.49, 1.56)	0.66	0.99 (0.54,1.83)	0.99
Q3	0.96 (0.54, 1.69)	0.89	1.24(0.68, 2.25)	0.49
Q4	**2.06 (1.29, 3.38)**	**0.003**	**3.21 (1.91, 5.51)**	**< 0.001**
**Nutrient-rich dietary factor**				
Q1	1.00		1.00	
Q2	0.89 (0.51, 1.54)	0.67	0.81 (0.45, 1.46)	0.49
Q3	0.89 (0.51, 1.54)	0.67	0.75 (0.41, 1.36)	0.34
Q4	**1.75 (1.10, 2.85)**	**0.02**	1.57 (0.95, 2.66)	0.09

Model 1 is unadjusted. Model 2 is adjusted for sex, ethnicity, marital status, household income, education, smoking status, alcohol consumption, and BMI.

## Discussion

This study contributes new evidence on dietary patterns and healthy aging in a Canadian population. The nutrient-rich dietary pattern was not associated with being a SS after adjustment for confounders, but the highest quartile of the western dietary pattern was associated with greater odds of being a SS. This was in contrast to our hypothesis as the western dietary pattern was predominately characterized by consumption of foods considered to be less healthy including processed meat, red meat, sauces and gravies, fried and non-fried potatoes, high sugar snacks, salty snacks and high fat dairy products. Nonetheless, it is important to note that the western dietary component contained other foods that did not meet our threshold for component loading (e.g. poultry and eggs) but may have still contributed to associations.

Consistent with findings from other studies of older adults including the Georgia Centenarian Study [21, 22], the Alameda County Study [23] and the Harvard College Alumni Study [23], SS engaged in health behaviors known to promote healthy aging [17]. Compared to CLSA participants, SS had a higher prevalence of never smokers. None were current smokers and few were obese, although habitual alcohol drinking was more prevalent among SS.

Our finding of an apparent disconnect whereby the less healthy western dietary pattern and the nutrient-rich dietary pattern were both associated with greater odds of being a SS (in unadjusted models) is similar to findings from the Georgia Centenarian Study [13, 24]. They reported centenarians consumed a more varied diet including higher consumption of milk and grains compared to adults in their 60’s. Conversely, centenarians were also more likely to consume whole milk and biscuits and less likely to follow nutrition guidelines for chronic disease risk reduction. The authors suggested that the differences in diet may reflect functional problems in the very old, for example oral health and chewing functions. We did not specifically query difficulty eating but the prevalence was likely low among SS as the inclusion criteria of no chronic diseases correlates with a generally healthy population, and the prevalence of meal replacements was low among SS (data not shown).

It is unclear whether the tendency of SS to follow a western-dietary pattern reflects generational and/or cohort differences with CLSA participants, longevity-related differences or possibly fatalism (e.g. approaching the end of one’s life) which has a negative impact on health behaviors [25, 26]. For example, higher frequency of high fat foods may reflect ingrained generational dietary patterns. National dietary guidelines were not significantly modified until the 1980’s in Canada to emphasize energy balance and moderation (limiting fat, sugar, salt and alcohol) [27]. A prior study by Takeda et al. [8] reported that dietary trends among two cohorts of centenarians studied in 1981 and 1995 differed with respect to cereal, egg, algae products and legume intake that were attributed to generational differences. There is also a potential influence of sociodemographic factors. Education was lower among SS and a higher proportion of SS lived alone, both of which are associated with poor diet [28]. Associations between the western-dietary pattern and SS remained with adjustment for both variables suggesting that marital status and education were not driving the observation.

There are several limitations to our study. While it is not practical to monitor food intake over the lifespan, current diets may not reflect past dietary habits that may be more likely to contribute to healthy aging. Appropriate comparisons for the oldest old are limited. The vast majority of individuals in the same birth cohort as the oldest old generally do not survive to an advanced age and those that do, have chronic disease may be unable to participate in research studies. Our use of the CLSA cohort from the same geographic areas as SS’s is the same approach used by centenarian studies [13, 24] but it also introduces potential generational and cultural influences. To minimize generational differences we included CLSA participants up to the oldest age range at the time of dietary assessment (age 86), however the majority of CLSA participants were from younger generations than SS; 188 (1.5%) were aged 85 and older. The SDQ was chosen as the dietary tool in our study to facilitate comparison to the CLSA. However, the SDQ was not designed to quantify the amount of foods consumed or nutrient intake, and is likely prone to recall bias inherent in self-reported assessment of dietary intake. Our results and inferences are thus limited to frequency of consumption rather than amount. This may be an important limitation as total food intake decreases with age [8]. Our sample size of 122 SS although large with respect to the rarity of this population is modest for characterizing dietary patterns, and generalizability to other populations is limited. Lastly, there are numerous factors related to dietary intake and healthy aging that were not measured including social factors, physical activity and genetic variation that would be informative to assess in future studies [29].

## Conclusion

In conclusion, our results suggest a western dietary pattern is associated with increased odds of healthy aging in this sample of older Canadian adults. This finding adds to the limited evidence base of dietary intake among the healthiest oldest old, but its implications should be considered in the context of possible generational differences in diet and limitations of the assessment of food and nutrient frequency but not amount of food.

## Supplementary information

**Additional file 1 Appendix Table 1.** Covariate estimates from logistic regression models estimating the odds ratio (OR) of being a super-senior (SS) participant for the western and nutrient-rich dietary factors among 12,626 CLSA and 122 SS

## Data Availability

The SS dataset is available from the corresponding author on reasonable request. The CLSA dataset is available are available from the CLSA but restrictions apply to the availability of these data, which were used under license for the current study and so are not publicly available. Data are however available from the authors upon reasonable request and with permission of the CLSA.

## Abbreviations

CCHS: Canadian Community Health Survey
CLSA: Canadian Longitudinal Study on Aging
FFQ: Food frequency questionnaire
OR: Odds ratio
Q: Quartile
SDQ: Short dietary questionnaire
SS: Super seniors
