# Supplementary material for: Dietary patterns in the healthy oldest old in the healthy aging study and the Canadian longitudinal study of aging: a cohort study
Source: BMC Geriatr. 2020 Mar 16;20:106. doi: 10.1186/s12877-020-01507-w (PMC7077120; doi:10.1186/s12877-020-01507-w)
Supplement: Supplementary file 1 — Additional file 1 Appendix Table 1. Covariate estimates from logistic regression models estimating the odds ratio (OR) of being a super-senior (SS) participant for the western and nutrient-rich dietary factors among 12,626 CLSA and 122 SS [file 12877_2020_1507_MOESM1_ESM.docx]

**Dietary patterns in the healthy oldest old in the Healthy Aging Study and the Canadian Longitudinal Study of Aging: a cohort study**

**Appendix Table 1.** Covariate estimates from logistic regression models estimating the odds ratio (OR) of being a super-senior (SS) participant for the western and nutrient-rich dietary factors among 12,626 CLSA and 122 SS

|  | **Model 2** | |
| --- | --- | --- |
| **Western dietary factor** | OR (95% CI) | p-value |
| Female | 1.06 (0.70-1.61) | 0.79 |
| Other ethnicity | 0.72 (0.12-2.36) | 0.65 |
| Living with a partner | 0.24 (0.15-0.37) | <0.001 |
| Income $50,000-$99,999 | 1.31 (0.82-2.07) | 0.25 |
| Income ≥$100,000 | 1.63 (0.89-2.92) | 0.11 |
| Education >high school & <Bachelor’s degree | 0.74 (0.45-1.22) | 0.23 |
| ≥Bachelor’s degree | 0.43 (0.26-0.72) | 0.001 |
| Former smoker | 0.67 (0.45-0.99) | 0.04 |
| Habitual alcohol consumption | 1.92 (1.05-3.71) | 0.04 |
| Occasional alcohol consumption | 0.86 (0.45-1.72) | 0.66 |
| Regular alcohol consumption | 0.69 (0.33-1.46) | 0.32 |
| Underweight BMI | 2.90 (1.06-6.67) | 0.02 |
| Overweight BMI | 0.28 (0.18-0.43) | <0.001 |
| Obese BMI | 0.07 (0.02-0.15) | <0.001 |
| **Nutrient-rich dietary factor** |  |  |
| Female | 0.81 (0.53-1.23) | 0.32 |
| Other ethnicity | 0.55 (0.09-1.79) | 0.41 |
| Living with a partner | 0.26 (0.16-0.40) | <0.001 |
| Income $50,000-$99,999 | 1.30 (0.82-2.06) | 0.27 |
| Income ≥$100,000 | 1.60 (0.86-2.88) | 0.13 |
| Education >high school & <Bachelor’s degree | 0.71 (0.43-1.16) | 0.17 |
| ≥Bachelor’s degree | 0.37 (0.22-0.61) | <0.001 |
| Former smoker | 0.68 (0.46-1.01) | 0.05 |
| Habitual alcohol consumption | 2.07 (1.14-4.00) | 0.02 |
| Occasional alcohol consumption | 0.87 (0.45-1.72) | 0.67 |
| Regular alcohol consumption | 0.73 (0.35-1.54) | 0.40 |
| Underweight BMI | 2.88 (1.06-6.60) | 0.02 |
| Overweight BMI | 0.29 (0.19-0.45) | <0.001 |
| Obese BMI | 0.07 (0.03-0.17) | <0.001 |
